# Genome-wide association mapping dissects the selective breeding of determinacy and photoperiod sensitivity in common bean (*Phaseolus vulgaris* L.)

**DOI:** 10.1093/g3journal/jkaf090

**Published:** 2025-04-19

**Authors:** Kate E Denning-James, Caspar Chater, Andrés J Cortés, Matthew W Blair, Diana Peláez, Anthony Hall, José J De Vega

**Affiliations:** Earlham Institute, Norwich, NR4 7UZ, UK; Royal Botanic Gardens, Kew, Richmond TW9 3AE, UK; Royal Botanic Gardens, Kew, Richmond TW9 3AE, UK; Department of Plants, Photosynthesis and Soil, School of Biosciences, The University of Sheffield, Sheffield S10 2TN, UK; Corporación Colombiana de Investigación Agropecuaria (AGROSAVIA)—C.I. La Selva, Rionegro 054048, Colombia; Facultad de Ciencias Agrarias—Departamento de Ciencias Forestales, Universidad Nacional de Colombia—Sede Medellín, Medellín, Colombia; Department of Agricultural and Environmental Sciences, College of Agriculture, Tennessee State University, 3500 John A Merritt Blvd, Nashville, TN 37209, USA; Corporación Colombiana de Investigación Agropecuaria (AGROSAVIA)—C.I. La Selva, Rionegro 054048, Colombia; Earlham Institute, Norwich, NR4 7UZ, UK; Earlham Institute, Norwich, NR4 7UZ, UK

**Keywords:** common bean, legume, determinacy, photoperiod, GWAS, domestication, Plant genetics and genomics

## Abstract

The common bean (*Phaseolus vulgaris* L.) is a legume pulse crop that provides significant dietary and ecosystem benefits globally. We investigated 2 key traits, determinacy and photoperiod sensitivity, that are integral to its management and crop production, and that were early selected during the domestication of both Mesoamerican and Andean gene pools. Still, significant variation exists among common bean landraces for these traits. Since landraces form the basis for trait introgression in prebreeding, understanding these traits’ genetic underpinnings and relation with population structure is vital for guiding breeding and genetic studies. We explored genetic admixture, principal component, and phylogenetic analyses using whole-genome sequencing to define subpopulations and gene pools. We used genome-wide association mapping (GWAS) to identify marker-trait associations in a diversity panel of common bean landraces. We observed a clear correlation between these traits, gene pool, and subpopulation structure. We found extensive admixture between the Andean and Mesoamerican gene pools in some regions. We identified 13 QTLs for determinacy and 10 QTLs for photoperiod sensitivity and underlying causative genes. Our study identified known and novel causative genes and a high proportion of pleiotropic effects for these traits in common bean, and likely translatable to other legume species.

## Introduction

The common bean is a global staple that provides significant dietary and economic services by improving health and nutrition while helping to reduce poverty, specifically in developing countries. Common beans have also been labeled as one of the essential crops to mediate climate change due to their lower environmental impact and protection of food and nutritional security (Foyer *et al*. 2016). Common beans are cultivated mainly as grain legumes, but the immature seeds, pods, and leaves are also eaten (Blair *et al*. 2010; Ganesan and Xu 2017). There are hundreds of varieties, and the prevailing type grown in a country depends on market preferences (Rawal and Navarro 2019). Common beans are rich in essential dietary components, such as protein, minerals, fiber, and micronutrients (Patto *et al*. 2015; Blair, Izquierdo, *et al*. 2013; Castro-Guerrero *et al*. 2016; Ganesan and Xu 2017), and protect against some forms of malnutrition, including stunting in children and micronutrient deficiencies (Jha *et al*. 2015; Suarez-Martinez *et al*. 2016; Ganesan and Xu 2017; Bernardi *et al*. 2023). As legumes, common beans have a symbiotic relationship with nitrogen-fixing bacteria, allowing them to fix atmospheric nitrogen and enhance nitrogen levels in the soil, thereby reducing the need for expensive chemical fertilizers while improving yields (Mylona *et al*. 1995; Cusworth *et al*. 2021; Mupangwa *et al*. 2021; Phiri and Njira 2023). Despite its widespread usability, trait segregation within and among bean landraces is still widespread, especially for critical agronomic traits such as growth habit and photoperiod.

The common bean underwent 2 separate domestications resulting in 2 gene pools: Andean and Mesoamerican. In addition, there are different races, intermediate species, and admixed accessions due to genetic isolation, fragmentation, and artificial selection for different morphological traits. The gene pools of common beans grow in a large variety of environments in the neotropics. These ecogeographic conditions, together with isolation by distance, have disrupted the gene flow between wild and domesticated common beans, and between the different gene pools (Santalla *et al*. 2004; Beebe *et al*. 2012). Consequently, there are large differences in their life history traits, morphology, and genetics (Gepts and Debouck 1991; Broughton *et al*. 2003; Beebe *et al*. 2012; Bitocchi *et al*. 2017). Another difference is cultivars are commonly autogamous and annual, while wild common beans and related species can be perennial and allogamous (Debouck *et al*. 1993; Schier *et al*. 2019; Chacon-Sanchez *et al*. 2021).

Photoperiod insensitivity and determinacy arose separately in both gene pools during the domestication of common beans, likely co-selected by growers (Weller *et al*. 2019; Repinski *et al*. 2012). Wild common beans tend to be indeterminate and photoperiod sensitive, requiring a particular day length to flower. Indeterminate growth is advantageous in the wild due to competition with surrounding vegetation, while photoperiod sensitivity (PS) was likely reinforced by divergent natural selection and local adaptation. On the other hand, photoperiod insensitivity was selected (likely unconsciously) as cultivated common beans were spread along a greater range of latitudes and environments. Determinacy, a developmental feature that causes common beans to have a terminal inflorescence when switching to a reproductive state (Cavalcante *et al*. 2020), optimized agricultural management and harvesting efficiency. Determinate common beans tend to have a bush growth habit with reduced branching and vining abilities compared with the indeterminate varieties (Kwak *et al*. 2012), therefore translocating biomass resources into an increased fitness output. While indeterminate and photoperiod sensitive landraces are common, the combined selection for photoperiod insensitivity and determinacy resulted in common bean varieties with shorter flowering periods, earlier maturation, and easier management during harvesting (Daba *et al*. 2016; González *et al*. 2016). Photoperiod insensitivity and determinacy are advantageous traits from an agronomical point of view due to earlier harvesting and shorter exposure to unfavorable weather patterns under climate change, consequently providing better food security for communities (Perez *et al*. 2020; Botero and Barnes 2022).

Modern breeding programs are moving beyond a yield-centered paradigm to target resistance to biotic and abiotic stress, and also nutritional quality (Singh and Schwartz 2010; Assefa *et al*. 2019; Caproni *et al*. 2020; Kachinski *et al*. 2022). Landraces and crop wild relatives offer a promising reservoir of genetic diversity for these traits by introgression from the landraces into the elite genetic background (Tai *et al*. 2014; Hu *et al*. 2021; Suarez, Polania *et al*. 2021; Suarez, Urban, *et al*. 2021). However, understanding the genetic diversity, population structure, patterns of adaptations, and how these correlate with determinacy and photoperiod insensitivity is required to guarantee the retention of these key domesticated traits within future breeding cycles, given their association with crop management and production (Beebe *et al*. 2012).

Common beans in Colombia are diverse regarding growth habits and PS. Colombia is the northernmost part of the Andean gene pool and south of the Mesoamerican and may act as a region of confluence between them. Consequently, it has been proposed that the region has a large amount of admixture and introgressive hybridization (Tohme *et al*. 1996; Blair *et al*. 2007; Blair, Cortes, *et al*. 2013; Leitao, Bicho, *et al*. 2021). Admixture and hybridization lead to introgressions from differential parental origins, introducing new alleles and novel epistatic interaction into a population, allowing for new trait combinations that could merge exotic variation from diverse germplasm with more agronomically desirable traits such as determinacy and photoperiod insensitivity.

Considering the above hypothesis, we characterized 144 representative landraces from Colombia and neighboring countries, together with controls from other regions, using whole-genome re-sequencing. We utilized genome-wide association mapping (GWAS) to identify significant SNPs for photoperiod insensitivity and determinacy in this diversity panel. The novelty of this work lies in that prior research commonly focused on the Mesoamerican diversity rather than the Andean, due to the greater genetic diversity in the former, and had ignored admixed materials as an essential source of variation. Furthermore, research has rarely utilized whole-genome sequencing of common bean accessions to undertake a GWAS on determinacy and photoperiod insensitivity phenotypes. Instead, previous work has mostly used QTL mapping and low-density marker panels, resulting in poor resolution (Kwak *et al*. 2008; González *et al*. 2016; García-Fernández *et al*. 2021).

## Materials and methods

### Diversity panel

**Fig. 1.**
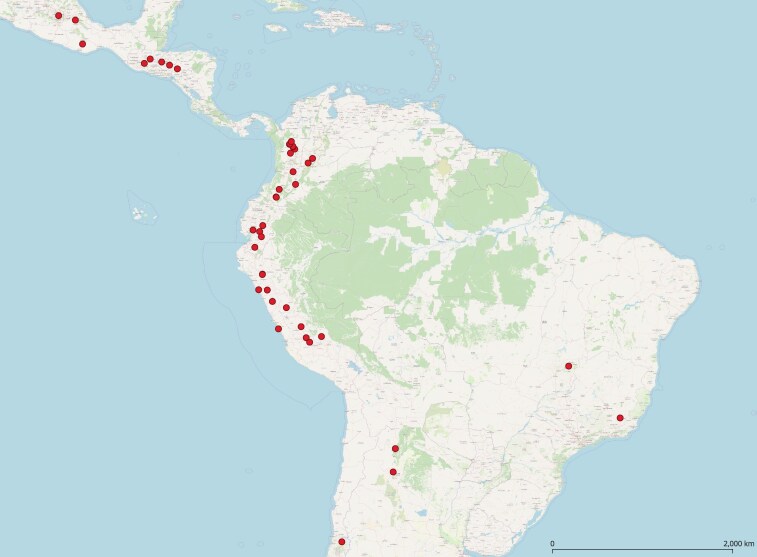
Distribution of the 127 common beans with location data that were used in this study. The coordinates of the capital city were used for those without coordinate data. Produced with QGIS.

The diversity panel was comprised of 144 genotypes mainly from Colombia and surrounding countries in Central and South America (Fig. 1). The panel contained accessions from elite backgrounds, landraces, heirlooms, weedy, and wild materials. The material was sourced from the International Centre for Tropical Agriculture (CIAT)'s genebank, the Leibniz Institute of Plant Genetics and Crop Plant Research (IPK)'s genebank, and heirlooms bought from the catalogs from “Jungle Seeds” (JungleSeeds 2020) and (Beans and Herbs 2020) in 2020. The panel was chosen to include control accessions from the Andean and Mesoamerican gene pools and races, while representing diverse seed coat colors and varying genetic backgrounds from Colombia and neighboring countries to focus on putatively admixed varieties.

### Genotyping

The genotypes were whole genome re-sequenced using Illumina short reads. The accessions were grown at the Norwich Research Park (Norwich, UK) in 2021 until the expansion of the first true leaf, after which they were snap-frozen (∼50–100 mg). The genomic DNA extraction for short-read sequencing from each accession was completed using a Qiagen DNAeasy kit (Qiagen, Germany). The DNA concentration of the samples was quantified for quality control using the Tecan Plate Read Infinite F200 Pro for a fluorometry-based assay. The sequencing of the samples was completed by Genomic services at Earlham Institute (Norwich, UK). LITE libraries, a cost-effective low-volume variant of the standard Illumina TruSeq DNA protocol, were constructed for the 144 accessions and were sequenced using 2 NovaSeq 6000 S4 v 1.5 flow cells with 150 bp paired-end reads, following the protocol in (Kirkwood *et al*. 2021).

### Phenotyping

All 144 common bean accessions were evaluated at the Norwich Research Park (Norwich, UK) in temperature-controlled glasshouses. The experiments were conducted in 2 seasons; summer 2022 with long daylength (16:8) and winter 2023 with short daylength (12:12). The accessions were organized in a randomized block design with 3 or 2 replications, respectively. Management was conducted according to recommendations for common bean cultivation.

The diversity panel was characterized for the days to flowering (DTF), seed size (SS), weight of 100 seeds (E100_SW; estimated based on the weights of seeds harvested and projected to 100 seeds), determinacy (D; terminal flower bud presence) (Cavalcante *et al*. 2020), and PS (flowering in none, 1 or both seasons). DTF was split into the 2 seasons due to PS in certain accessions and PS was characterized in 3 ways for the GWAS.

The statistical analysis of variance (1-way ANOVA) of the phenotypic data was done in R, then the Pearsons's correlation coefficient was calculated and visualized using the R package “corrplot” (Wei and Simko 2021).

### Preprocessing genotype data

The raw sequence reads were processed with TrimGalore (v. 0.5.0) (Krueger *et al*. 2023) to remove adapters and poor-quality reads, and then quality checked using FastQC (Wingett and Andrews 2018) and MultiQC (Ewels *et al*. 2016). The trimmed reads were aligned to the Andean reference genome, *Phaseolus vulgaris* G19833, v2.1 (Schmutz *et al*. 2014) downloaded from Phytozome (Goodstein *et al*. 2012) with BWA-MEM (v 0.7.13) (Li and Durbin 2009) and “-M -R” to add read group information and allow compatibility with GATK. SAMtools (v 1.7) combined, compressed, and sorted the aligned files (Danecek *et al*. 2021). Picardtools (https://broadinstitute.github.io/picard/) (v 2.1.1) marked duplicates and BamTools indexed the alignments (Barnett *et al*. 2011). The percentage of alignments were calculated at this stage. The genotype data were divided into 10 Mbp regions (Garrison and Marth 2012) (v 1.0.2) to run the Genome Analysis ToolKit (GATK v 4.2) haplotype caller with default parameters (Van der Auwera and O'Connor 2020). This identified 20.2 million variant loci (∼17.1 M SNPs and ∼3.4 M indels).

### Population structure analysis

The resulting VCF file from GATK using the Andean reference (“Andean VCF”) was filtered further with BCFtools to retain calls with a minimum depth of 5 reads per variant call (FMT/DP ≥ 5), a locus call quality over 30, maximum missing calls per locus of 5%, to keep only biallelic SNP locus, and for a minor allele frequency over 2%. The resulting VCF had ∼9 million SNP loci. Then, the VCF was filtered for a maximum heterozygosity of 20% per locus using TASSEL 5 (v. 20230314) (Bradbury *et al*. 2007). This was then filtered for linkage disequilibrium (LD) (based on LD decay) and thinned with a window size of 10 bps using BCFtools prune.

The population structure of the panel was analyzed using ADMIXTURE (v 1.3.0) (Alexander and Lange 2011) on a subset of 88,786 SNP loci. ADMIXTURE was run for K = 2 to K = 10 and the ideal number of K was determined using the cross-validation error. Accessions were allocated a group when their membership coefficient (q) was greater than 0.7. Plotting was completed in R using the packages “ggplot2’ (Ginestet 2011).

### Genome-wide association study

The “Andean VCF” from GATK was filtered with BCFtools (v 1.12) (Danecek *et al*. 2021) for biallelic loci, a minor allele frequency of 1% and thinned with a window size of 5 bp. To understand the genetic relationship between accessions, we used a principal component analysis (PCA) generated with GAPIT v.3 (Wang and Zhang 2021) on a subset of 2,572,124 loci.

A genome-wide association study investigated marker-trait association for determinacy and photoperiod insensitivity phenotypes using GAPIT v.3 (Wang and Zhang 2021) with 3 principal components. We ran with the models Bayesian-information and Linkage-disequilibrium Iteratively Nested Keyway (BLINK) (Huang *et al*. 2019), Fixed and random model Circulating Probability Unification (FarmCPU) (Liu *et al*. 2016), and Mixed Linear Model (MLM) (Zhang *et al*. 2010). BLINK and FarmCPU were identified as the best multi-locus models for different heritability levels, improving statistical power (Huang *et al*. 2019; Merrick *et al*. 2022; Cebeci *et al*. 2023). While MLM was chosen for single-locus analysis as a baseline for comparison to BLINK and FarmCPU.

GAPIT was run on the whole panel (144 accessions) and on the Andean subpanel (as defined at K2 ADMIXTURE; 108 accessions). To run BLINK, GAPIT completed the analysis with the option “Random.model = TRUE” as not to calculate R^2^ for phenotypic variance explained values after GWAS. The quantile-quantile (QQ) plots were used to understand the suitability of the models to the data. Plotting was completed in R using the package “ggplot2’ (Ginestet 2011).

### Selecting significant loci, candidate gene mining, and functional annotation

Significant marker-trait associations (MTAs) were investigated further when they had a −log_10_(*P*-value) over 7 and were confirmed by 2 models from GAPIT. QTLs were defined as ±100 kbp from the MTA based on the estimated LD decay distances in common bean diversity panels and by using a r^2^ = 0.25 cutoff (estimated decay as 114 kb) (Moghaddam *et al*. 2016; Valdisser *et al*. 2017; Campa *et al*. 2018; Raggi *et al*. 2019; Wu *et al*. 2020, 2024; Ugwuanyi *et al*. 2022; Reinprecht *et al*. 2023). This is shorter than the calculated recombination rate in common bean of 3.72 cM/Mb (Bhakta *et al*. 2015). LD decay was estimated for the diversity panel (mean R^2^ = 0.27) and subpopulation at K = 2 (Andean mean R^2^ = 0.21, Mesoamerican mean R^2^ = 0.2) using PopLDdecay software following Wu *et al*. (2020) (Zhang *et al*. 2019).

Identified loci were compared with the Andean reference genome, *Phaseolus vulgaris* G19833 v2.1 in JBrowse (Schmutz *et al*. 2014; Diesh *et al*. 2023) while considering “highimpact” mutations identified by SnpEff (Cingolani *et al*. 2012). Once genes were identified, their putative function was explored using PhytoMine (Goodstein *et al*. 2012) (*Phaseolus vulgaris v.2*), BLAST (Camacho *et al*. 2009) against the nonredundant protein database at NCBI, and finally against the TAIR database if no gene function could be identified in close relatives (Huala *et al*. 2001). The loci were compared with previous studies and literature. PulseDB was used for comparison, particularly for QTLs and markers related to developmental and flowering phenotypes (Humann *et al*. 2019). QTLs and markers were mapped to the reference genome to estimate the conversion from cM to Mb in JBrowse.

## Results

### Population structure

**Fig. 2.**
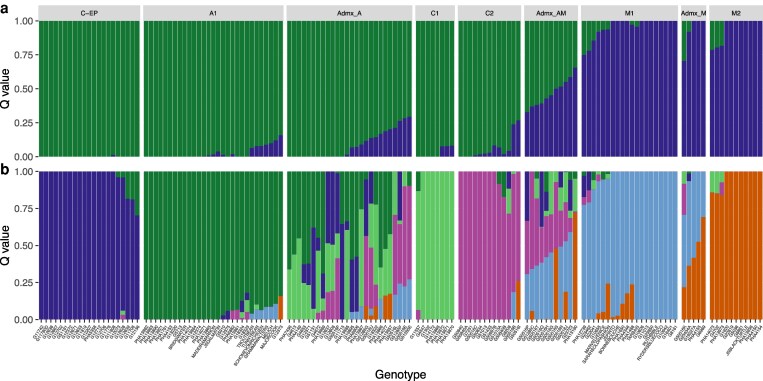
Analysis of the population structure of 144 accessions belonging to our diversity panel focusing on Colombia at K = 2, Andean or Mesoamerican groups a) and K = 6 b). (C-EP) accessions mainly from Peru, then Ecuador and Colombia; (A1) Andean accessions from a variety of South American countries; (C1) mostly determinate Colombian landraces; (C2) indeterminate Colombian landraces; (M1) mainly medium seeded** from Central America and Colombia; (M2) mainly small seeded** from Central America and Colombia. (Admx_AM) Andean X Mesoamerican hybrids; (Admx_A) and (Admx_M) admixed accessions between subpopulations (ancestry composition q < 0.7 at K = 6). **P < 0.01 using a 2-tailed student *t*-test with unequal variance.

**Fig. 3.**
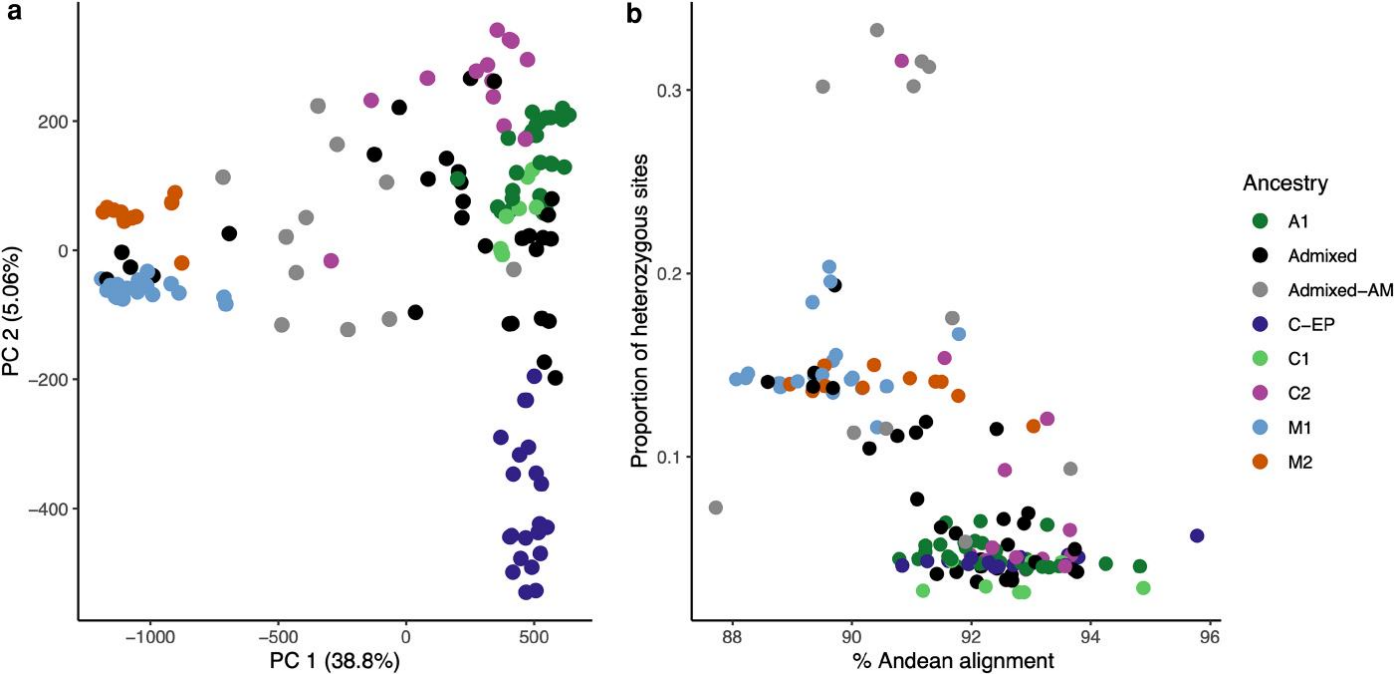
a) Principle component analysis (PCA) plot of PC1 against PC2. b) Proportion of heterozygous sites against the percentage of read pair alignment to the Andean reference genome G19833 (Schmutz *et al*. 2014). The colors illustrate the population structure of our diversity panel.

The diversity panel split into the 2 gene pools, the Andean and Mesoamerican (Figs. 2a and 3a). At K6 (Fig. 2b), the Mesoamerican group split into 2 subpopulations (M1 and M2), while the Andean subgroup split into 4 subpopulations. Two of these subpopulations included only accessions from Colombia and were named C1 and C2. A subpopulation containing accessions from Colombia and Ecuador/Peru was named C-EP. The remaining subpopulation was named A1. In the PCA (Fig. 3a), PC 1 explained 38.8% of the variation in our diversity splitting the 2 gene pools, while PC2 accounted for 5.06% of the variation, splitting the Mesoamerican subgroups (M1 and M2) and separating C-EP from the other Andean subgroups. A total of 11 accessions were classified as admixed between the Andean and Mesoamerican gene pools (Admx_AM), as they had an ancestry composition lower than 70% from either of the origins (q < 0.7). The Admx_AM accessions were all indeterminate and produced a variety of seed sizes. Seven were landraces and 2 were wild. There was also a mix of photoperiod sensitive and insensitive accessions.

The Colombian subgroups (C1 and C2; Fig. 2b) contained medium and large seeded landraces. However, the subpopulations distinguished by determinacy; C1 contained mainly insensitive determinate accessions while C2 contained sensitive indeterminate accessions. The A1 group contained large and medium seeded landraces that were mainly photoperiod insensitive. The C-EP population contained accessions from Ecuador, Peru, and Colombia. This group contained large-seeded indeterminate landraces and also included accessions from races previously identified to be from the Andean gene pool. The Mesoamerican subgroups (M1 and M2; Fig. 2b) were also distinguished by phenotypic data. They both contained indeterminate and determinate accessions; however, M1 was mainly medium seeded while M2 was mainly small seeded. This is summarized in Table 1 and Supplementary Table 1.

**Table 1.** Phenotypic characteristics associated with each subpopulation.

Subpopulation	Gene pool	Determinancy	Photo. sen.	Seed size	Origin
C1	Andean	Mainly determinate	Insensitive	Mainly large	Colombia and Ecuador
C2	Andean	Indeterminate	Mainly sensitive	Mainly large	Colombia
A1	Andean	Both	Mainly insensitive	Mainly large	South America, Heirlooms, Colombia
C-EP	Andean	Indeterminate	Sensitive	Large	Colombia, Ecuador, Peru
Admix_A	Andean	Mainly indeterminate	Both	Mainly large	Colombia and South America
M1	Mesoamerican	Mainly indeterminate	Both	Mainly medium**	Central America, Colombia, Heirlooms, Peru
M2	Mesoamerican	Mainly indeterminate	Mainly insensitive	Mainly small**	Central America, Colombia
Admix_M	Mesoamerican	Mainly indeterminate	Insensitive	Small and medium	Colombia, Brazil, Heirlooms, Central America
Admix_AM	AxM hybrids	Indeterminate	Mainly sensitive	Mainly medium	Colombia and Ecuador

Colombian accessions can be found within all the subgroups and admixed groups at K = 6 (Fig. 2b). While the admixture accessions are mainly from Colombia, while 1 sample is a wild “Ecuador” accession.

The Andean accessions had a lower proportion of heterozygous sites (<0.1) than the Mesoamerican accessions, which were more heterozygous (Fig. 3b). The 6 highly heterozygous accessions (>25% of the loci) were found within the Andean X Mesoamerican hybrid (Admixed-AM) subpopulation (Fig. 3b) and were from Colombia. Finally, the outlier accession with the lowest alignment to the Andean reference genome and low proportion of heterozygous sites was a wild accession from Ecuador.

### Phenotypic variation and correlations

**Fig. 4.**
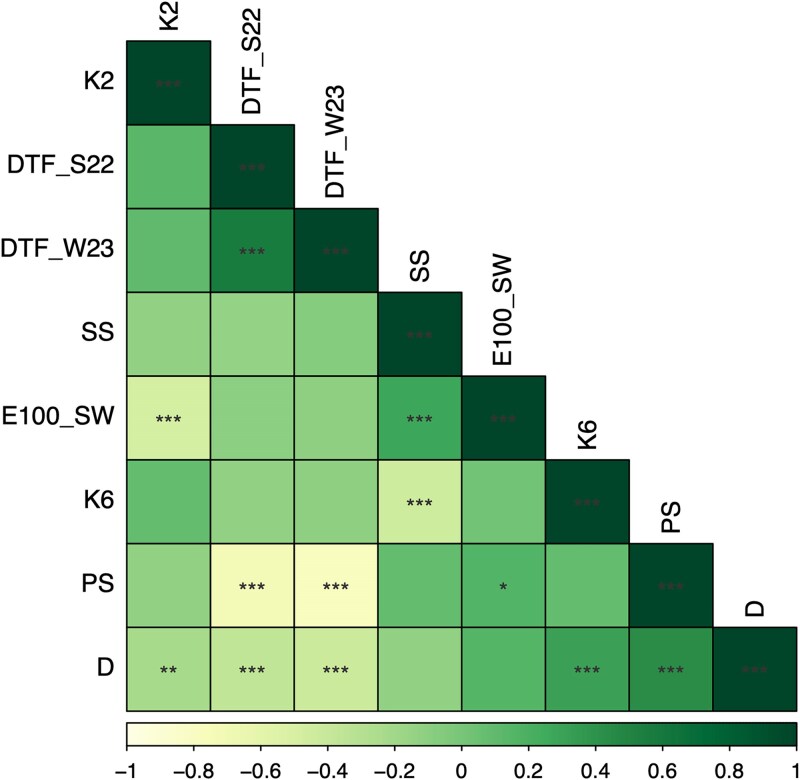
Pearson correlation coefficients among five agronomic traits and population structure measured in 144 common bean genotypes grown at the Norwich Research Park, Norwich, UK in 2022 and 2023. K6, K6 subgroups from ADMIXTURE; K2, K2 subgroups from ADMIXTURE; D, determinacy; PS, photoperiod sensitivity; SS, seed size; E100_SW, estimated weight of 100 seeds; DTF_W23, DTF from winter 2023; DTF_S22, DTF from summer 2022. *P < 0.05; **P < 0.01; ***P < 0.001.

The correlation coefficient was estimated for each pair of traits (Fig. 4), averaged over 2 seasons or studied in both years. There was a positive correlation between DTF from winter and summer (r = 0.57). Both DTF were negatively correlated with PS [r = −0.72 (DTF_S22), r = −0.77 (DTF_W23)] and D [r = −0.35 (DTF_S22), r = −0.43 (DTF_W23)]. Population structure at either 2 or 6 ancestries (K2, K6) was positively correlated with D [r = 0.32 (K6), r = 0.37 (K2)] but negatively correlated with SS [r = −0.44 (K6), r = −0.4 (K2)] and E100_SW [r = −0.37 (K6), r = −0.47 (K2)]. SS was not correlated with DTF_S22, DTF_W23, D, or PS (r = −0.13, r = −0.07, r = −0.12, r = 0.09). However, E100_SW was positively correlated with PS (r = 0.18) and SS (r = 0.87) but negatively correlated with DTF_S22 (r = −0.22). Then D and PS were positively correlated (r = 0.45).

**Fig. 5.**
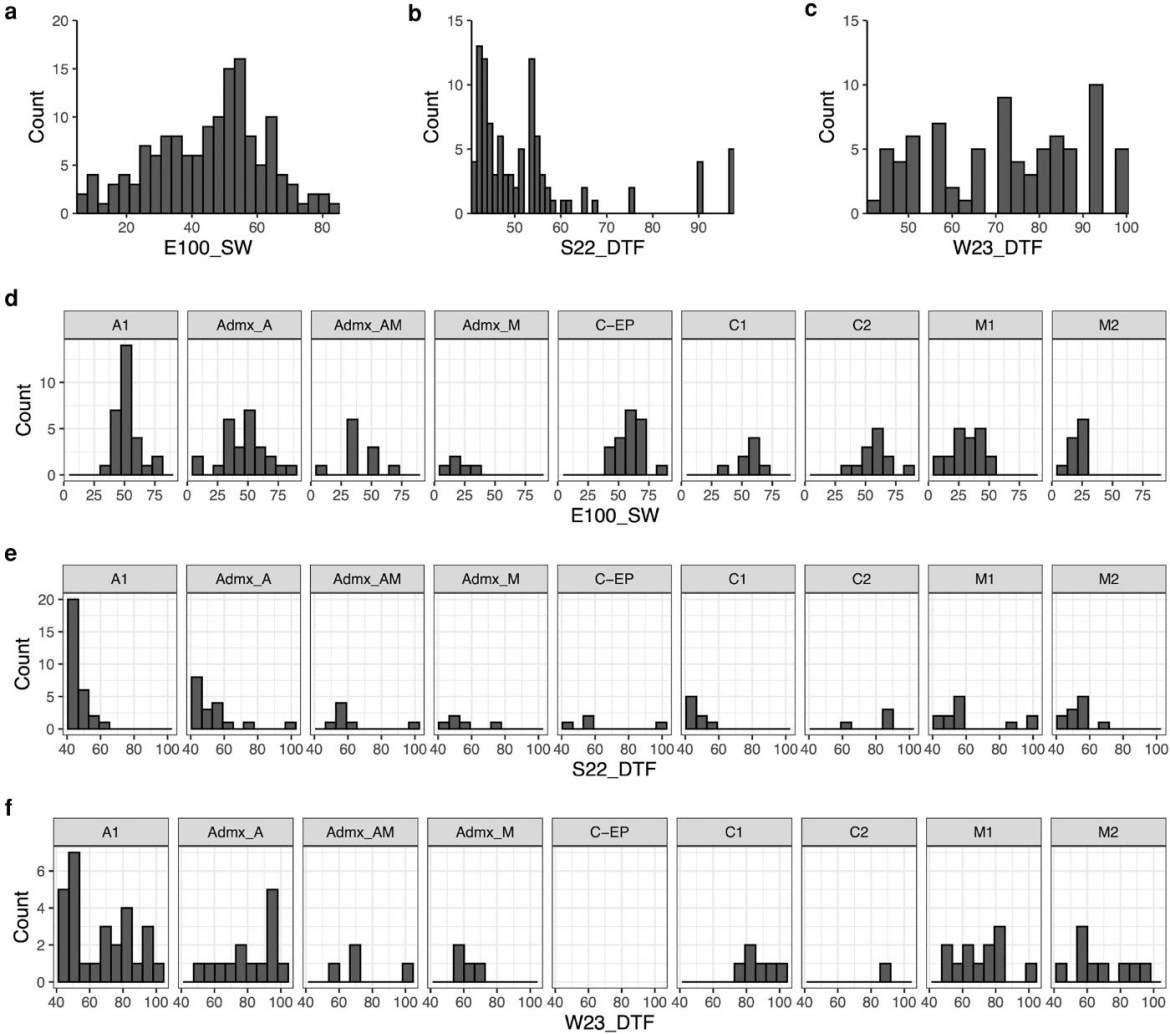
Frequency distribution of seed weight and days to flower traits evaluated in 2 seasons in a common bean diversity panel. a) E100_SW, estimated weight of 100 seeds; b) phenological DTF in the summer 2022 (S22_DTF) and c) in the winter 2023 (W23_DTF) at the Norwich Research Park, excluding those which did not flower. The distributions were split into the subpopulations from K6 ADMIXTURE. d) E100_SW***; e) S22_DTF***; f) W23_DTF*. Completed a 1-way ANOVA for E100_SW, S22_DTF, and W23_DTF. *P < 0.05; **P < 0.01; ***P < 0.001.

Figure 5, a–c showed the distributions of the phenotyping for traits E100_SW, S22_DTF, and W23_DTF, respectively. The seed weights (Fig. 5a) were normally distributed, while the DTF in summer and winter (Fig. 5, b and c) were binomial distributions; the peaks were around 42- and 54-days postsowing in summer, and around 70- and 90 days in winter. When analyzing the phenotypes by subpopulation, we can see that C-EP (Fig. 2b) did not flower during winter in the UK, W23_DTF, as was mainly photoperiod sensitive. This is further supported by the correlation plot (Fig. 4). Furthermore, determinacy, photoperiod insensitivity, and DTF are correlated. The determinate accessions flower earlier than the indeterminate, supporting the binomial distribution.

### GWAS for determinacy

**Fig. 6.**
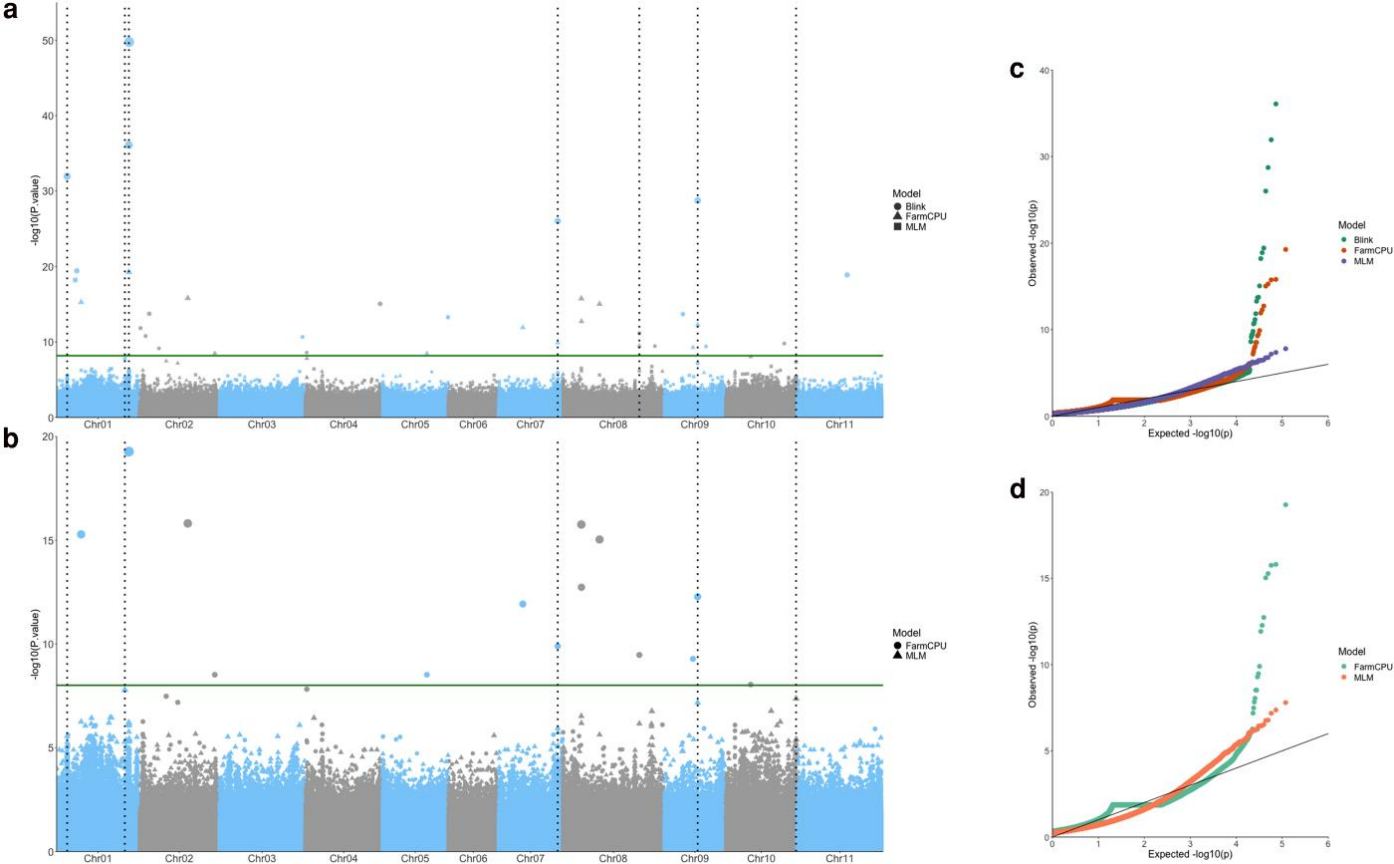
Manhattan plots highlighting markers significantly associated with determinacy on (a) the whole panel and b) the Andean subpanel. The analyses were completed with GAPIT and the models are FarmCPU, BLINK, or MLM (Huang *et al*. 2019; Liu *et al*. 2016; Wang and Zhang 2021; Zhang *et al*. 2010). The *X*-axis represents the genomic position of markers and the *Y*-axis is the −log 10 of the *P*-values for association with the phenotype. The vertical lines correspond to QTLs found by at least 2 models. Point size correlates to −log10(*P*-value). Quantile-quantile (QQ) plots are provided for c) the whole panel and d) the Andean panel.

The GWAS was performed using the models BLINK, FarmCPU, and MLM with GAPIT (Fig. 6, a and b). The QQ plots (Fig. 6, c and d) provided evidence that the selected models were well fitted to identify significant MTAs for the dataset. We identified 13 MTAs with a significant *P*-value (−log_10_(*P*-value) > 7), corresponding to 13 QTLs. We focused on 7 significant MTAs that were identified for the whole panel based on the criteria laid out in the methods (vertical lines in Fig. 6). The 7 QTLs were found on chromosomes Pv01, Pv07, Pv08, Pv09, and Pv10 (Table 2). Five of the 7 QTLS were also identified for the Andean subset.

**Table 2.** QTLs for determinacy and photoperiod sensitivity.

Name	Chromosome	Start	End	Trait	Panel
D1.1	Chr01	6,512,000	6,521,000	Determinacy	Andean + Whole
D1.2	Chr01	11,363,000	11,372,000	Determinacy	Andean
D1.3	Chr01	42,404,000	42,413,000	Determinacy	Andean + Whole
D1.4	Chr01	44,856,000	44,847,000	Determinacy	Whole
D1.5	Chr01	44,932,000	44,941,000	Determinacy	Andean + Whole
D1.6	Chr01	45,098,000	45,107,000	Determinacy	Whole
D2.1	Chr02	24,821,000	24,830,000	Determinacy	Andean
D3.1	Chr03	25,608,000	25,617,000	Determinacy	Andean
PS4.1	Chr04	38,316,000	38,325,000	Photo sensitivity	Whole
PS5.1	Chr05	16,423,000	16,432,000	Photo sensitivity	Whole
PS5.2	Chr05	18,321,000	18,330,000	Photo sensitivity	Andean
PS7.1	Chr07	16,829,000	16,838,000	Photo sensitivity	Andean + Whole
PS7.2	Chr07	26,485,000	26,494,000	Photo sensitivity	Andean + Whole
D7.1	Chr07	36,860,000	36,869,000	Determinacy	Andean + Whole
PS8.1	Chr08	4,234,000	4,243,000	Photo sensitivity	Whole
D8.1	Chr08	7,440,000	7,449,000	Determinacy	Andean
PS8.2	Chr08	8,320,000	8,329,000	Photo sensitivity	Andean
D8.2	Chr08	47,582,000	47,591,000	Determinacy	Whole
D9.1	Chr09	20,814,000	20,823,000	Determinacy	Andean + Whole
PS9.1	Chr09	21,640,000	21,649,000	Photo sensitivity	Whole
PS9.2	Chr09	34,445,000	34,454,000	Photo sensitivity	Andean
D10.1	Chr10	43,762,000	43,771,000	Determinacy	Andean + Whole
PS11.1	Chr11	204,000	213,000	Photo sensitivity	Andean

Putative candidate genes were identified for determinacy based on the significant MTAs and corresponding QTL windows. The identified genes and QTLs are listed in Supplementary Tables 2 and 3.

### GWAS for PS

**Fig. 7.**
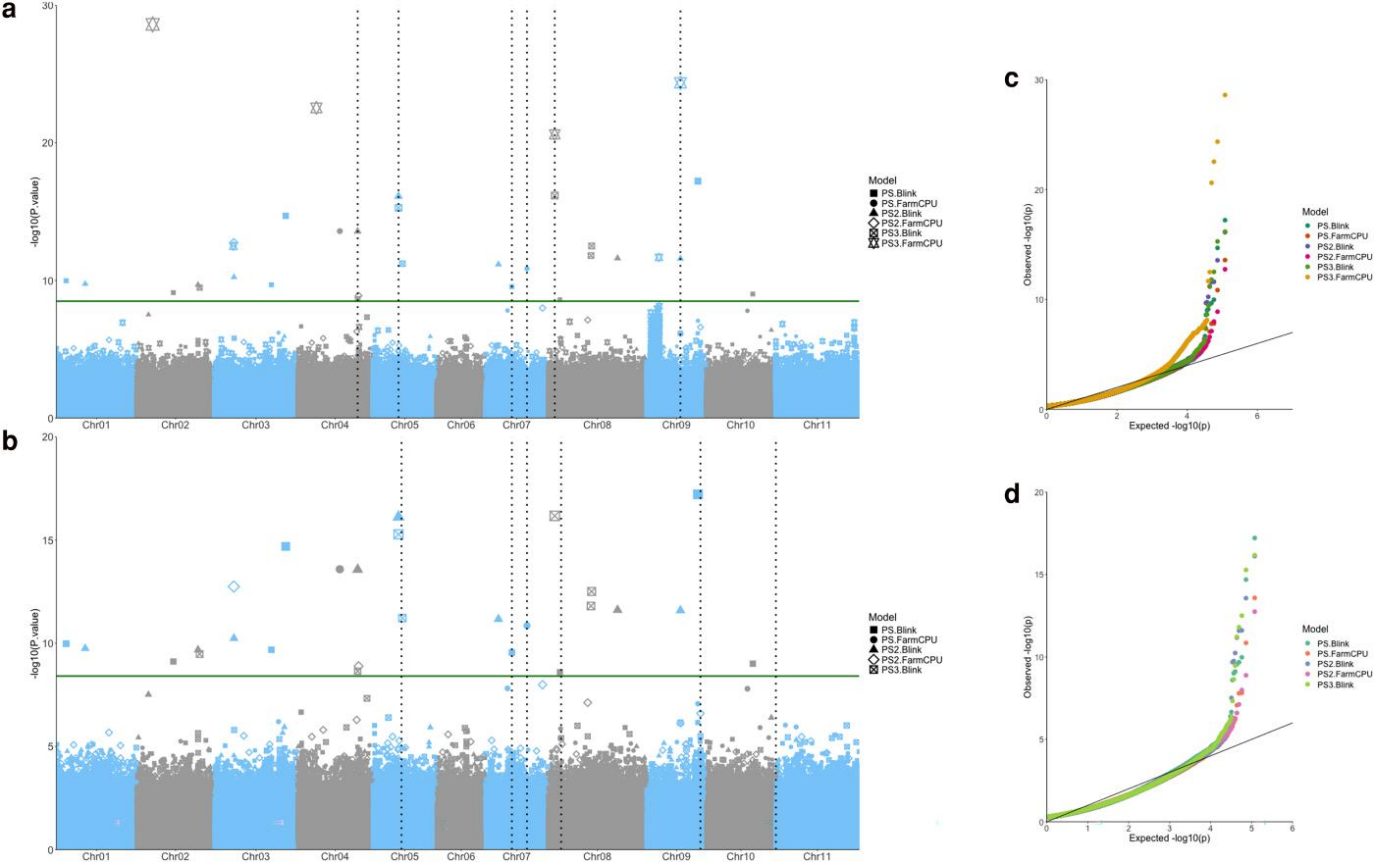
Manhattan plots highlighting markers significantly associated with photoperiod insensitivity on (a) the whole panel and b) the Andean subpanel. The analyses were completed with GAPIT and the models FarmCPU, BLINK, or MLM (Zhang *et al*. 2010; Liu *et al*. 2016; Huang *et al*. 2019; Wang and Zhang 2021). The *X*-axis represents the genomic position of markers and the *Y*-axis is the −log 10 of the *P*-values for association with the phenotype. The vertical lines correspond to QTLs found by at least 2 models. Point size correlates to −log10(*P*-value). Quantile-quantile (QQ) plots are provided for c) the whole panel and d) the Andean panel.

The GWAS was performed using the BLINK and FarmCPU models with GAPIT (Fig. 7, a and b). The QQ plots (Fig. 7, c and d) provide evidence that the selected models are fitted to identify significant MTAs for the dataset. We identified 10 QTLs (-log_10_(*P*-value) > 7). We focused on 6 QTLs for the whole panel based on criteria laid out in the methods. The MTAs were found on chromosomes Pv04, Pv05, Pv07, Pv08, and Pv09 (vertical lines in Fig. 7). Six QTLs were identified for the Andean subset panel in Chromosomes Pv05, Pv07, Pv08, Pv09, and Pv11. The QTL in Pv04 and Pv09 were found in the full dataset only. The QTL in Pv9 and Pv11 were found in the Andean subset only. Candidate genes were identified for the significant MTAs and their corresponding QTLs. The identified genes and QTLs are listed in Supplementary Tables 2 and 3.

## Discussion

We delimited subpopulations in a panel of 144 accessions, initially divided by domestication event into the 2 Andean and the Mesoamerican gene pools (Figs. 2 and 3) (Blair, Cortes, *et al*. 2013; Kami *et al*. 1995). The Mesoamerican gene pool is generally more diverse (Mamidi *et al*. 2013; Schmutz *et al*. 2014) with less influence from domestication bottlenecks. Furthermore, the Mesoamerican gene pool within our diversity panel is also more heterozygous, suggesting that the Andean gene pool has undergone fewer outcrossing events. These crosses between gene pools occur during common bean dissemination, breeding programs and selection based on market preferences (Hoyos-Villegas *et al*. 2017; de Almeida *et al*. 2020; Botero *et al*. 2021; Bellucci *et al*. 2023). However, care needs to be taken when utilizing market sampling information. This is highlighted by the 2 “Peruvian” accessions collected from markets that fall with the Mesoamerican subpopulation (Supplementary Table 1).

Admixture was commonly observed in the panel, including 26 admixed Andean accessions, 5 admixed Mesoamerican accessions, and 11 Mesoamerican × Andean accessions. This supports our initial hypothesis that Colombia and neighbouring countries hold large common bean variation, including hybrids between both gene pools (Gori *et al*. 2022; Myers *et al*. 2000; Pironon *et al*. 2020). The wider crosses between gene pools compared with within gene pools resulted in a larger observed heterozygosity in the hybrid accessions, supporting the outcrossing events and movement between gene pools. One implication of this study is that admixed Colombian hybrid landraces bridge Andean and Mesoamerican gene pools, and novel allelic and epistatic interactions likely filtered out deleterious effects (Cichy *et al*. 2015) due to stronger purifying selection with increased recombination. After all, recombination increases local effective population size (Ne) and limits Hill–Robertson interference (Hill and Robertson 2007). This suggests the Colombian hybrids have promising potential for breeding. However, the diversity panel may also be biased and underestimating their prevalence in other regions due to the large number of Colombian accessions in our diversity panel.

We observed some traits associated with demography, including determinacy and PS: C1 and C2 shared origin but could be separated by ancestry admixture analysis, and were characterized by different determinacy, as C1 contained mainly determinate accessions, and C2 mainly indeterminate accessions. Furthermore, the population structure suggests that Colombian farmers have not selected varieties based on the seed characteristics studied (e.g. SS) (Botero *et al*. 2021).

Indeterminate and photoperiod sensitive landraces were common, despite the combined selection for photoperiod insensitivity and determinacy resulting in common bean varieties with shorter flowering periods (DTF) and easier management. Prior research supports the correlation between DTF and phenotypes such as seed weight, determinacy and growth habit (Tar'an *et al*. 2002; Moghaddam *et al*. 2016; Hoyos-Villegas *et al*. 2017; Elias *et al*. 2021; Vargas *et al*. 2021). These phenotypes are related to apical meristems and floral development (Sablowski 2007).

We observed the distribution of DTF values, in either summer or winter, were bimodal, i.e. had 2 peaks (Fig. 5, b and c). This likely occurred due to the determinate types flowering first and then followed by the indeterminate beans (Coelho *et al*. 2023). The distribution also correlates to growth habits as bush types typically flower earlier than climbing types (Ugwuanyi *et al*. 2022). Figure 2a supports that PS arose during domestication in both gene pools (Weller *et al*. 2019).

The Andean accessions within our diversity panel were large and medium seeded while the Mesoamerican accessions were small and medium sized, which supports previous research (Blair *et al*. 2009). Among the Mesoamerican accessions, the Mesoamerican race is characterized by small-seeds, while the Durango–Jalisco race is characterized by medium seeds (Beebe *et al*. 2000; Zhang *et al*. 2008; Blair *et al*. 2009; Giordani *et al*. 2022). We could not separate our diversity panel into subpopulations matching these races due to a lack of Mesoamerican diversity in the panel, a limited genetic component for the SS trait, or introgressions occurring in the Mesoamerican Colombian accessions.

Interestingly, Ecuador accessions are often separated from Andean subgroups, suggesting that they are members of the PhI group or a possible sister species *Phaseolus debouckii* (Chacon-Sanchez *et al*. 2007; Rendon-Anaya *et al*. 2017). Further to this, the wild Ecuador accession is separated from both gene pools (Figs. 2 and 3), suggesting a separate ancestry originating from Ecuador or Peru (Bitocchi *et al*. 2012; Bitocchi *et al*. 2017). Finally, the C-EP group (Fig. 2b) are mainly photoperiod sensitive (Fig. 5f), possibly due to a different domestication history or due to their quatorial provenance not necessitating evolution under fluctuating photoperiods.

By leveraging this diversity panel and its trait segregation across the demographic stratification, we prioritized 13 QTLs for determinacy and 10 QTLs for PS. Four of the QTLs for PS, and 4 for determinacy, were also identified only for the Andean subset, but not the whole panel. The Andean gene pool has adapted to lower latitudes than the Mesoamerican pool, resulting in differential selection for PS between the 2 gene pools. The LD was estimated as 114 kb from an R^2^ cutoff of 0.25, this value is consistent with WGS data of diversity panels rather than breeding populations (Campa *et al*. 2018; Diniz *et al*. 2018; Reinprecht *et al*. 2023; Ambachew *et al*. 2024). LD in common beans is impacted by the evolutionary and breeding history of the accessions in the diversity panel; therefore, a 200 kb region accounts for the higher resolution of WGS as well as allowing for LD (Moghaddam *et al*. 2016; Valdisser *et al*. 2017).

During this study we completed analysis with the Andean reference genome (Schmutz *et al*. 2014). This reference genome was selected for being the most complete at the time of analysis and because our panel has a higher proportion of Andean accessions based on population structure analysis (Fig. 2). The accessions also had higher alignments to the Andean reference genome (92.5% ± 1 and 89.9% ± 1.1% for the Andean and Mesoamerican subpopulations, respectively) and no difference in metrics to the Mesoamerican reference genomes (Supplementary Table 1).

### QTLs and candidate genes associated with determinacy

#### Three QTLs in chromosome 1

We identified a determinacy QTL in chr 1 -Pv01- (D1.4-D1.6; Table 2), identified in other studies (Moghaddam *et al*. 2016; da Silva *et al*. 2018; Kamfwa *et al*. 2019; Sedlar *et al*. 2020; Vargas *et al*. 2021; Keller *et al*. 2022) as a hotspot of allelic variation, named the *Fin* locus. The *Fin* locus has been mapped to ∼44.5 Mb (Pérez-Vega *et al*. 2010; Kamfwa *et al*. 2019). This co-segregates with an upstream gene, *TFL1y* (*Phvul*.001G189200), a candidate gene for flowering, vegetative growth, rate of plant production, and determinacy (Kwak *et al*. 2008, 2012; Repinski *et al*. 2012; Cichy *et al*. 2015; González *et al*. 2016; Campa *et al*. 2018; Delfini *et al*. 2021). Consequently, the *Fin* locus has pleiotropic effects due to associations with many development traits such as determinacy, shoot biomass, DTF, days to maturity, plant architecture, embryo abortion, number of pods per plant, number of seeds per plant (seed yield and weight), and disease resistance (Miklas *et al*. 2001; González *et al*. 2016; Delfini *et al*. 2021; Soler-Garzón *et al*. 2024). However, segregation for this QTL hotspot in Pv01 may prove difficult in breeding programs due to these pleiotropic effects (Vargas *et al*. 2021).

Further candidate genes have been identified in this QTL, such as *Phvul.001G192200*. This gene is an ortholog of *LIGHT-REGULATED WD1* (*LWD1*), a gene involved in the circadian rhythm pathway (Wu *et al*. 2008; Moghaddam *et al*. 2016; Delfini *et al*. 2021), or *Phvul.001G192300,* which is an ortholog of *SPINDLY* (*SPY*). *SPY* interacts with genes in the reproductive pathway (Tseng *et al*. 2004; Moghaddam *et al*. 2016; da Silva *et al*. 2018) and has been associated with days to maturity (Reinprecht *et al*. 2023).

Another QTL we identified on Pv01 (D1.3; Table 2) contains the gene *Phvul.001G168700*. This gene is related to the phytochrome interacting factor 1 (PIF1) transcription factor isoform X1 in the legume *Vigna radiata* (Bateman *et al*. 2023). This bHLH transcription factor is involved in many light-dependent pathways in plant development and interacts with circadian clock genes (Kim *et al*. 2016).

#### QTL D7.1 in chromosome 7

The QTL at Pv07 (D7.1) was identified in the whole and Andean panel. The QTL contains the gene *Phvul.007G244700.* This is related to a transcriptional corepressor, Leunig-homolog in *Vigna radiata* (Bateman *et al*. 2023). In *Arabidopsis*, Luenig-homologs have functional redundancy with Leunigs (LUGs), and are involved in embryo and floral development (Sitaraman *et al*. 2008). This QTL has been associated with SS, seed weight, and growth habit (Kwak *et al*. 2008; da Silva *et al*. 2018; Elias *et al*. 2021; Keller *et al*. 2022), suggesting it may have pleiotropic effects.

#### QTL D8.2 in chromosome 8

The QTL identified on Pv08 (D8.2; Table 2) for determinacy has previously been identified for plant architecture (da Silva *et al*. 2018). However, no gene with a clear function was identified. We have, however, identified a possible candidate gene for further investigation; *Phvul.008G170000.* This encodes a putative fantastic 4 (FAF) domain-containing protein. In *Arabidopsis,* FAF proteins regulate shoot meristem size and architecture (Wahl *et al*. 2010).

#### QTL D9.1 in chromosome 9

The QTL D9.1 in chr 9 was identified in the whole and Andean panel. Nearby QTLs have been identified for yield and determinacy (Kamfwa *et al*. 2015; Campa *et al*. 2018). The gene *Phvul.009G138100* is found within this QTL and contains the significant MTA found by GAPIT (Wang and Zhang 2021). This gene has an insertion that possibly affects function (Cingolani *et al*. 2012). This gene is uncharacterized in common bean but has homology to the root meristem growth factor 9 from *Glycine soja* (Goodstein *et al*. 2012; Bateman *et al*. 2023). This growth factor is expressed in the roots and flowers, regulating and maintaining apical meristems, and therefore both root and floral development, SS, and leaf architecture (Chen *et al*. 2019; Shinohara 2021). Although it has previously been identified as a candidate gene associated with Mesoamerican domestication (Schmutz *et al*. 2014), we found the QTL in the Andean panel, suggesting that it has also played a role in the Andean domestication event.

#### QTL D10.1 in chromosome 10

The QTL on Pv10 (D10.1) is located near QTLs for plant height and number of nodules and near genes associated with metabolic changes during domestication, once again suggesting pleiotropic effects (Delfini *et al*. 2021; de Souza *et al*. 2023). Three of the genes within this region encode bHLHLZip proteins: *Phvul.010G158500, Phvul.010G158300*, and *Phvul.010G158200.* These bHLH transcription factors may be involved in the regulation of flowering genes (Zhou *et al*. 2019). The gene *Phvul.010G158500* displays nonsynonymous modifications in our panel, including insertions, deletions, and other variants linked to frameshift mutations and gained stop codons (Cingolani *et al*. 2012). Homology to *Vigna angularis* suggests this gene may be related to the transcription factor bHLH25, and possibly linked to a circadian rhythm-associated protein (Goodstein *et al*. 2012).

### Candidate genes for PS

#### QTL PS4.1 in chromosome 4

One QTL for PS was found on Pv04 (PS4.1; Table 2) from the analysis on the whole panel. Within this QTL, 4 genes were identified, 3 of which (*Phvul.004G110200*, *Phvul.004G110301*, and *Phvul.004G110000*) have nonsynonymous mutations such as a stop lost, stop gained, or a frameshift mutation in our panel (Cingolani *et al*. 2012). However, the genes are uncharacterized.

#### Two QTLs in chromosome 5

Two QTLs were identified in Pv05: PS5.2 for the Andean panel and PS5.1 for the whole panel. PS5.2 overlaps with a previously identified QTL for seed weight, DTF, and pod weight (Arriagada *et al*. 2022; Reinprecht *et al*. 2023). However, this previous analysis with a limited number of markers did not identify a candidate gene. Based on sequence homology with *Vigna radiata,* we identified the gene *Phvul.005G077000*, which encodes a proton gradient regulation 5 (PGR5) protein (Bateman *et al*. 2023). PGR5 is involved in plant growth under different light conditions due to interactions with Photosystem I, and consequently putatively associated with differentiating PS in our panel (Munekage *et al*. 2002). The QTL PS5.1 contained 2 genes, one of which, *Phvul.005G076300*, may encode a bidirectional sugar transporter, named SWEET protein. Evidence suggests SWEET proteins have essential roles in plant development, including in reproductive organs and bud growth (Gautam *et al*. 2022).

#### Two QTLs in chromosome 7

Two QTLs were also identified on Pv07. PS7.1 and PS7.2, both in the Andean and the whole panel. The QTL PS7.2 contains the genes *Phvul.007G157400* and *Phvul.007G156200*. Homology with *Arabidopsis* suggests that *Phvul.007G157400* encodes a BANQUE3 BHLH161 protein. BANQUE3 is negatively regulated by *APETALA3* and *PISTILLATA* in petals and is involved in light-regulated responses and flowering time (Huala *et al*. 2001; Mara *et al*. 2010). *Phvul.007G156200* may encode the BHLH transcription factor PIF4 (Phytochrome Interacting Factor 4) based on homology with *Vigna radiata* and *Glycine soja* (Goodstein *et al*. 2012; Bateman *et al*. 2023). PIF4 is a downstream signaling component integrating environmental cues such as light (Bateman *et al*. 2023).

The QTL PS7.1 overlaps with a previously identified QTL for plant production traits (González *et al*. 2016). The QTL includes the gene *Phvul.007G117400* which encodes a putative JUMONJI domain-containing protein (Goodstein *et al*. 2012). JUMONJI proteins are involved in multiple plant developmental processes such as flowering and leaf senescence (Gan *et al*. 2014; Liu *et al*. 2019; Yamaguchi 2021; Xin *et al*. 2024). *Phvul.007G117400*s homology with a JUMONJI16 orthologue in *Vigna radiata* also supports this role (Bateman *et al*. 2023).

#### Two QTLs in chromosome 8

One of the QTLs found in Pv08 is PS8.1 from the whole panel. This QTL has been associated with determinacy (Campa *et al*. 2018), seed weight (Elias *et al*. 2021), DTF (Raggi *et al*. 2019), and pod number (Kamfwa *et al*. 2015). Due to the marker technology used, the QTL for seed weight was large so had low resolution (Elias *et al*. 2021). Our results (Fig. 4) suggest a correlation between DTF, determinacy, and PS under the same QTL. The significant MTA for this QTL was within the gene *Phvul.008G048300*. However, the function of this gene is currently unclear.

The other QTL found on Pv08 is PS8.2, which has previously been identified for seed weight (Blair *et al*. 2006). Genes within this QTL include *Phvul.008G085000, Phvul.008G084500, Phvul.008G084900*, and *Phvul.008G084100*. *Phvul.008G085000* is homologous to *gibberellin 2-oxidase 8* in *Arabidopsis* (Huala *et al*. 2001). Gibberellin oxidases may respond to light intensity, and can therefore be related to PS (Zhang *et al*. 2022). *Phvul.008G084100* is homologous to *CLAVATA3* in *Arabidopsis,* a gene that regulates shoot and floral meristem development (Clark *et al*. 1995; Hirakawa 2021). *Phvul.008G084900* is homologous to genes encoding ovate family proteins (OFPs). OFPs appear to be sensitive to light stimuli (Shahzaib *et al*. 2024). *Phvul.008G084500* has homology with *RAVEN/INDETERMINATE DOMAIN5* in *Arabidopsis,* which is linked to GA signaling pathways as well as other plant developmental pathways (Sanchez-Corrionero *et al*. 2019; Aoyanagi *et al*. 2020). *Phvul.008G085000* and *Phvul.008G084900* also both contain insertions or deletions with high-impact nonsynonymous mutations which, therefore, possibly affect function (Cingolani *et al*. 2012).

#### Two QTLs in chromosome 9

A QTL was identified on Pv09 in the Andean panel (PS9.1). This was near a QTL associated with grain yield (Elias *et al*. 2021), postharvest index (Sedlar *et al*. 2020), shoot biomass (Kamfwa *et al*. 2019), SS (da Silva *et al*. 2018), DTF, and yield (Blair *et al*. 2006). Genes within the QTL included *Phvul.009G229100, Phvul.009G229200, Phvul.009G229700*, and *Phvul.009G229900. Phvul.009G229100* is homologous to PIN3 transcription factor genes, involved in regulating root and shoot growth (Goodstein *et al*. 2012; Haga and Sakai 2012). Homology with *Arabidopsis* suggests *Phvul.009G229200* and *Phvul.009G229700* are involved in root growth (Huala *et al*. 2001), and that *Phvul.009G229900* encodes a *HAB1 (Hypersensitive To Aba1) homology to ABI (Abscisic Acid-Insensitive)1* gene involved in ABA signal transduction, which is regulated by circadian rhythm (Leitao, Santos, *et al*. 2021; Kamrani *et al*. 2022). The other QTL in PV09 (PS9.2) was found in the whole panel and included the gene *Phvul.009G145100*, which was also related to an ABA response gene in *Arabidopsis*. A nearby QTL to PS9.2 was previously identified for DTF (Keller *et al*. 2022).

#### QTL PS11.1 in chromosome 11

The QTL at PV11 (PS11.1) was near a QTL for seed weight (da Silva *et al*. 2018) and a QTL for disease resistance (Banoo *et al*. 2020). This may be due to pleiotropic effects or low resolution of the previous analysis with a limited number of markers. Within this QTL is the gene *Phvul.011G004000* which encodes a putative PHD finger protein. PHDs have been found to be involved in the regulation of flowering time (Zhou *et al*. 2019; Qian *et al*. 2021). Other genes within the QTL are related to root or shoot growth. For example, homology of *Phvul.011G003200* and *Phvul.011G003400* implicates them in processes involved in root meristem development (Huala *et al*. 2001). *Phvul.011G003700* is an uncharacterized gene in common bean but homology with *Arabidopsis* suggests it may be associated with phytochrome interacting factor 7 (PIF7) to regulate hypocotyl elongation (Huala *et al*. 2001; Leivar *et al*. 2008). However, there are many genes within this QTL and further research is needed to clearly distinguish a candidate gene.

## Conclusion

Our common bean panel contains genetic diversity from the Andean (4 subgroups) and Mesoamerican (2 subgroups) gene pools. Including accessions from Colombia that contain introgressive hybridization and admixture diversity from the Andean and Mesoamerican gene pools. There was a systematic association between the population structure and agronomic traits such as determinacy and PS. In this study we identified genomic regions which are connected to known and novel putative candidate genes involved in developmental and reproductive pathways. We found 13 QTLs associated with determinacy and 10 QTLs associated with PS. One known QTL was the *Fin* locus on Pv01 for determinacy known for its pleiotropic effects in plant development. While other putative candidate genes were identified due to homology with *Glycine soja, Vigna* species and *Arabidopsis.* This includes *Phvul.008G170000* that encodes a putative FAF domain-containing protein. Consequently, GWAS are important in identifying MTAs and candidate genes, especially when accounting for population structure. By linking candidate genes to phenotypes, we hope more targeted precision breeding approaches can be adopted to improve common bean traits under climate change. Nevertheless, this current study and previous ones highlight that for some genes and genomic regions, this will be difficult due to the high proportion of pleiotropic effects in common beans.

## Supplementary Material

jkaf090_Supplementary_Data

## Data Availability

We thank CIAT's Genebank and IPK's Genebank for their generous provision of germplasm. Germplasm held in the CIAT and IPK collections is available on request. Raw reads are deposited in the SRA under accession PRJEB81566. The scripts used in this study are publicly available in Github (https://github.com/DeVegaGroup/KDJ-CBeans/). Supplemental material available at G3 online.
