# Correction to: Genome-wide association mapping dissects the selective breeding of determinacy and photoperiod sensitivity in common bean (*Phaseolus vulgaris* L.)

**DOI:** 10.1093/g3journal/jkaf141

**Published:** 2025-06-24

This is a **correction** to:

Kate E Denning-James, Caspar Chater, Andrés J Cortés, Matthew W Blair, Diana Peláez, Anthony Hall, José J De Vega, Genome-wide association mapping dissects the selective breeding of determinacy and photoperiod sensitivity in common bean (*Phaseolus vulgaris* L.), *G3 Genes|Genomes|Genetics*, Volume 15, Issue 6, June 2025, jkaf090, https://doi.org/10.1093/g3journal/jkaf090

In the originally published version of this manuscript, the second affiliation for author, Andrés J Cortés, was translated from Spanish to English.

The correction affiliation should read:

Facultad de Ciencias Agrarias – Departamento de Ciencias Forestales, Universidad Nacional de Colombia – Sede Medellín, Medellín, Colombia

This error has been corrected.

