# Supplementary material for: Genome-wide association mapping dissects the selective breeding of determinacy and photoperiod sensitivity in common bean (Phaseolus vulgaris L.)
Source: G3 (Bethesda). 2025 Apr 19;15(6):jkaf090. doi: 10.1093/g3journal/jkaf090 (PMC12135006; doi:10.1093/g3journal/jkaf090)
Supplement: jkaf090_Supplementary_Data [file jkaf090_supplementary_data.zip › Supplementary_Table_Legends_G3-2025-405670.docx]

**Supplementary Table Legends**

**Supplementary Table 1.** Subpopulation, alignment and phenotypic annotation for the common bean accessions used in this study.

**Supplementary Table 2.** Genes and non-synonymous genetic variants within each of the identified QTLs associated with determinacy.

**Supplementary Table 3.** Genes and non-synonymous genetic variants within each of the identified QTLs associated with photoperiod sensitivity.
